# Evolution of Asthma Severity in a Cohort of Young Adults: Is There Any Gender Difference?

**DOI:** 10.1371/journal.pone.0007146

**Published:** 2009-09-25

**Authors:** Chantal Raherison, Christer Janson, Deborah Jarvis, Peter Burney, Lucia Cazzoletti, Roberto de Marco, Françoise Neukirch, Benedicte Leynaert

**Affiliations:** 1 Department of Respiratory Diseases Haut-Lévèque CHU Bordeaux, EA 3672, ISPED, Université Bordeaux 2, Bordeaux, France; 2 Department of Medical Sciences: Respiratory Medicine and Allergology, Uppsala University, Uppsala, Sweden; 3 Department of Public Health Sciences, Capital House, London, United Kingdom; 4 Unit of Epidemiology and Medical Statistics, Department of Medicine and Public Health, University of Verona, Verona, Italy; 5 Epidémiologie des Maladies Respiratoires, INSERM U700, Faculté de Médecine Xavier, Bichat, Paris; Helmholtz Zentrum München/Ludwig-Maximilians-University Munich, Germany

## Abstract

**Introduction:**

Little is known about the distribution of asthma severity in men and women in the general population. The objective of our study was to describe asthma severity and change in severity according to gender in a cohort of adult asthmatics

**Methods:**

Subjects with asthma were identified from random samples of the 22 to 44 year-olds from the general population, screened for asthma from 1991 to 1993 in 48 centers from 22 countries and followed-up during 1998–2002, as part of the European Community Respiratory Health Survey (ECRHS). All participants to follow-up with current asthma at baseline were eligible for the analysis. To assess change over the follow-up, asthma severity at the two surveys was defined using standardized data on respiratory symptoms, lung function and medication according to the Global Initiative for Asthma (GINA) Guidelines. Another quantitative score (Ronchetti) further considering hospitalizations was also analysed.

**Results:**

The study included 685 subjects with asthma followed-up over a mean period of 8.65 yr (min 4.3-max 11.7). At baseline, asthma severity according to GINA was distributed as intermittent: 40.7%, 31.7% as mild persistent, 14% as moderate persistent, and 13.5% as severe persistent. Using the Ronchetti score derived classification, the distribution of asthma severity was 58% mild, (intermittent and mild persistent), 25.8% moderate, and 15.4% severe. Whatever the classification, there was no significant difference in the severity distribution between men and women. There was also no gender difference in the severity distribution among incident cases which developed asthma between the two surveys. Men with moderate-to-severe asthma at baseline were more likely than women to have moderate-to-severe asthma at follow-up. Using GINA, 69.2% of men vs. 53.1% of women (p = 0.09) with moderate-to-severe asthma at baseline were still moderate-to-severe at follow-up. Using Ronchetti score, 53.3% of men vs. 36.2% of women (p = 0.03) with moderate-to-severe asthma at baseline were still moderate-to-severe at follow-up.

**Conclusions:**

There was no gender difference in asthma severity at the two surveys. However, our findings suggest that asthma severity might be less stable in women than in men.

## Introduction

Several studies have reported a higher severity of asthma in women than in men, both in the use of health care and in admissions to hospital [1]. In a population-based study, women had a 70% higher risk than men of being admitted to hospital for asthma after controlling for asthma prevalence and smoking [2]. Gender differences in hospital admissions for asthma could relate to differences in the disease severity, perception and management. In a cross-sectional study from the European Network For Understanding Mechanisms Of Severe Asthma (ENFUMOSA), women were found to dominate the group of subjects with severe asthma as compared to the patients whose asthma was controlled by low doses of inhaled corticosteroids[3]. In a high-risk adult asthmatic cohort, two thirds of admissions were women, suggesting that women might suffer from a more severe form of asthma [4]. Women also report more dyspnoea [5].

In a population-based prospective cohort with a 25-year follow-up [6], women were more likely than men to have severe asthma (OR: 1.57[1.19–2.08])), especially if asthma had developed after the age of 2 years and was associated with reduced PEF.(Peak Expiratory Flow).

In a cross-sectional survey, asthma severity increased with BMI (Body Mass Index)only in women [7]. In a retrospective analysis, it has been suggested that late-onset asthma, which generally occurs during or after puberty, affects mainly women, and has a poor prognosis[8]. In an unselected birth cohort, female sex predicted persistence of asthma. In addition, lung function in males in whom asthma relapsed after remission closely resembled that in males with persistent asthma, whereas females with a relapse had worsened lung function only as adults[9]. To our knowledge, no longitudinal population-based study has investigated gender difference in asthma severity.

The purpose of this study was to examine whether the evolution of asthma severity was different in women and in men in a longitudinal population-based survey.

## Methods

### Study design

The methods of the survey have been fully described elsewhere[10], [11]. Briefly, at the baseline survey (ECRHS I), 48 centers in 22 countries randomly selected around 3000 men and 3000 women aged 20 to 44 year-old who completed a short postal questionnaire about asthma and asthma-like symptoms (stage 1). From 1991 to 1993, a 20% random subsample of responders were invited to attend a local test center (stage 2) to complete a more detailed questionnaire administered by an interviewer and to undergo skin prick and blood tests, assessment of lung function by spirometry, and airway challenge with methacholine. The selection of these subjects was ideally made by random selection from a suitable sampling frame. The aim was to obtain 300 of each gender. In addition, participants who were not in the random sample, but who reported that they had at least one asthma-like symptom (symptomatic sample) or were currently taking medicine for asthma, were also invited to participate in stage 2. Participants gave written informed consent, and institutional or regional ethics committee approved the study in each participating center.

All participants to ECRHS I stage2 were eligible for participating in the follow-up survey (ECRHS II, n = 14 countries) during 1998–2002. The same protocol was used for the follow-up survey, including an administered clinical interview, lung function measurements, and serum IgE samples.

Twenty-seven centers participated in ECRHS II. The full protocol can be found at www.ecrhs.org.

### Definition of asthma

Participants were defined as having current asthma if they answered positively to the question “have you ever had asthma” and if they had had at least one asthma attack in the last 12 months or were “currently taking any medicines including inhalers, aerosols or tablets for asthma”. Subjects with current asthma at the baseline survey, but without current asthma at the follow-up survey were considered to be in remission at follow-up. Subjects who had no history of asthma in ECRHS-I and who had current asthma in ECRHS-II were considered to be incident asthmatics.

Only asthmatics whose respiratory function met the American Thoracic Society (ATS) criterion for reproducibility [12]were included in the study.

### Evaluation of asthma severity

Asthma severity was first classified as intermittent, mild persistent, moderate persistent, or severe persistent using the GINA guidelines[13] that were available at the time of ECRHS-I. Two independent GINA classifications were combined as recommended for the description of asthma severity in a population [14]one based on symptoms and FEV1 and called “Clinical Step” (Step C 1–4); and the other based on current medication and called “treatment step” (Step T 1–4) to construct a final “symptom-FEV1 medication” classification.

- GINA classification1992

Four levels of severity were considered for each of the “Clinical” and “Treatment” classifications (step)

**Clinical step (Step C)**

Step C1 (Intermittent): Asthma attacks less than once a week and FEV1≥80% predicted. The patients not classified as step 1 were assigned to the other steps as follows.
Step C2 (Mild persistent): all patients not allocated to the other steps, with a FEV1> = 80% predicted.
Step C3 (Moderate persistent): Patients with 60%< FEV1<80% predicted, or daily attacks
Step C4 (Severe persistent): All patients with FEV1< = 60% predicted.

**Treatment step (Step T)** (defined according to current asthma medication)

Step T1: No controller medication or only short-agonist bronchodilator.
Step T2: Inhaled corticosteroids or theophylline without oral corticosteroids
Step T3: Inhaled corticosteroids and theophylline or long-acting bronchodilator but without oral corticosteroids.
Step T 4: Oral corticosteroids.

For each patient, the final severity step was defined as the highest step between the “clinical step” and “treatment step”.

Subjects with asthma at ECRHS II were classified using the same classification, as for ECRHS I. However we also classified subjects with asthma at ECRHS II using a more detailed version of GINA (2004) that also takes into account the nocturnal symptoms, and the dose of inhaled steroids

- **GINA guidelines 2004** [**15**] were applied to the second survey and then compared with the 1992 guidelines in order to evaluate the agreement between the two classifications. The main differences were the presence of nocturnal symptoms for clinical step and inhaled corticosteroids for treatment step. This information was not available in the first survey.

Again, two independent GINA classifications were combined: one based only on symptoms and FEV1 and called “clinical step” (Step C 1–4); and the other based on current medication to construct a final “symptom-FEV1 medication” classification and called “treatment step” (Step T 1–4). Each step was divided into four levels of severity.

**Clinical step**

Step C 1 (Intermittent): symptoms less than once a week and nocturnal symptoms not more than twice a month and FEV1> = 80% predicted. The patients not classified as step 1 were assigned to the other steps as follows.
Step C 4 (Severe persistent): FEV1< = 60% predicted.
Step C 3 (Moderate persistent): Patients with 60%< FEV1<80% predicted, and daily symptoms or night-time asthma>1/week.
Step C 2 (Mild persistent): all patients not allocated to the other steps, with a FEV1> = 80% predicted.

**Treatment step** according to current asthma medication

Stept T1: No controller medication, or only short-agonist bronchodilator.
Stept T4: Oral corticosteroids or inhaled corticosteroids >2,000 ug/day
Stept T2: Inhaled corticosteroids < = 500 ug/day without oral corticosteroids
Stept T3: Inhaled corticosteroids< = 1000 ug/day and long-acting bronchodilator but without oral corticosteroids.

The final severity step was based on the two independent classifications of clinical step and treatment step, according to the GINA, as previously mentioned.

#### Ronchetti Score

Secondly, asthma was classified as intermittent, mild persistent, moderate persistent, severe persistent by using a second approach based on a score derived from Ronchetti et al.[16], and previously used in the same population[17]. This score was based on

- FEV1 (forced expiratory volume in one second) (mild>80%, moderate 70–80%, severe <70%),
- number of asthma attacks in the previous 12 months (2, 3–6, >6)
- number of admissions to hospital for breathing problems in the previous 12 months (0, 1–2, >2)
- wether inhaled or oral corticosteroids had been taken in the past 12 months.

Each of the first three variables had three levels of increasing severity (scored 1, 2 or 3) and the fourth variable had two levels (scored 1 or 2). The overall total score therefore ranged from 4 to 11, with levels of severity being intermittent (score 4 or 5, without inhaled corticosteroids), mild persistent (score 4 or 5, with inhaled corticosteroids), moderate persistent (score = 6) and severe persistent (score> = 7).

This classification was applied using the same algorithm at the two study periods.

### Statistical analysis

The associations between the different levels of severity of asthma and categorial variables were assessed with the test in the SAS-PC statistical package (SAS Institute, Cary, NC). The Kappa test was used to compare one classification used for severity versus another classification. 0.7 shoud be consider as a good concordance; 0.8–1 to an excellent concordance. Comparisons were done using synthesis of each classification.

Secondly, we performed logistic regressions to assess the odds ratio for the associations between risk factors and moderate-severe asthma versus intermittent-mild asthma, taking potential confounders into account, and geographic centers included in the model as an additional explanatory variable. Risk factors were selected among risk factors usually published in the literature, mainly age, sensitization to cat allergen, house dust mite, and molds, smoking habits. The logistic regression was a stepwise approach. Alpha risk was 5%.

## Results

**Figure 1:**
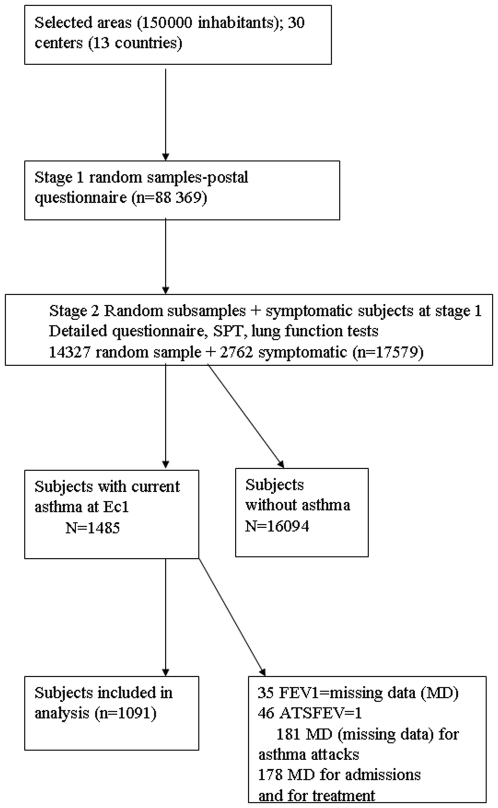
Study design.

All current asthmatics identified in ECRHS I and who participated in ECRHS II were eligible for this analysis. Of the 17579 participants examined at stage 2, 1485 currently had asthma in ECRHS-I. Of those, 1091 could be classified for asthma severity at Ec1 (missing data: 35 for FEV1, 46 had FEV1 value without ATS conformity, 181 for the number of asthma attacks, 178 for both admissions and asthma treatment) (figure 1).

In men (n = 481):

- 183 were lost to follow-up at Ec2
- 150 were in remission at Ec2
- 148 were still asthmatic at Ec2

In women (n = 610):

- 194 were lost to follow-up at Ec2
- 215 were in remission at Ec2
- 201 were still asthmatic at Ec2

Comparison between participants and subjects lost to follow-up are shown in table 1. The mean follow-up was 8.65 yr (min 4.3-max 11.7). There were significantly more females and smokers in the second sample. Moreover, there was no significant difference according to FEV1%pred, atopy or severity of asthma. Stratification by gender in men and women demonstrated a high prevalence of smokers in the sample “lost to follow-up” (table 1).

**Table 1:** Characteristics of subjects who provided respiratory data in the two assessments as compared with those not followed-up at the second survey by gender.

Gender	Men	Men		Women	Women	
	Participants	Lost to follow-up	p	Participants	Lost to follow-up	P
	N = 148	N = 183		N = 201	N = 194	
Age, m	33.3(7.2)	32.1(7.2)	0.36	34(7.2)	32.2(7.2)	0.36
Age of first asthma attack	14.3(11)	14(11.2)	0.23	19.3(11.7)	18.4(10.8)	0.13
Smoking, %	23.2	37.1	0.005	26.7	36	0.0037
%FEV1 pred	92.5(17.7)	91.7(18.7)	0.10	96.8(15.8)	94(17.2)	0.12
Atopy,%	66.3	68.9	0.56	58.2	61.3	0.48
Rhinitis,%	70.5	74.8	0.30	76	66.5	0.01
***Severity*** **,%**						
Intermittent	47.6	45.2	0.75	33.9	31.5	0.07
Mild P.	29.2	27.1		34.3	36.2	
Moderate P.	11.9	10.5		16.1	15.3	
Severe P.	11.3	11.2		15.8	14.7	
Hospitalizations,%	19.8	20.9	0.77	19.9	22.7	0.43
Emergency visits,%	32	34	0.63	34.5	33.	0.71

According to the 1992 GINA classification, 40.7% of asthmatics at baseline were classified intermittent, 31.7% as mild persistent, 14% as moderate persistent, and 13.5% as severe persistent. The comparison of men and women in EC1 by GINA (p = 0.04) is weak (table 2).

**Table 2:** Cross-sectional distribution of asthma severity in subjects with data available at both surveys.

	**EC1**	**EC1**	**P**	**EC2**	**EC2**	**P**
**“Ronchetti”**	Men	Women		Men	Women	
Mild, %	56.9	60.5	0.38	59.1	64.7	0.26
Moderate, %	28.7	22.9		21.1	20.3	
Severe, %	14.4	16.5		19.8	15	
	**EC2**	**EC2**	**P**	**EC2**	**EC2**	**P**
**“GINA”**	Men	Women		Men	Women	
I, %	47.6	33.9	0.04	28.8	28.5	0.89
II, %	29.2	34.3		37.6	35.1	
III, %	11.9	16.1		22.1	24.7	
IV, %	11.3	15.8		11.5	11.7	

* ***comparison of men and women in EC1 by GINA (p = 0.04) is weak.***

Using the Ronchetti score derived classification, the distribution of asthma severity was 58% mild, (intermittent and mild persistent), 25.8% moderate, and 15.4% severe.

Whatever the classification used, the distribution of asthma severity at baseline was not different in men and in women. At follow-up there was still no gender difference in the distribution of severity.

In subjects included in the second survey (n = 685, both followed and incident), the concordance of the two classifications was compared (GINA 1992 vs GINA 2004) at follow-up. The Kappa coefficient was 0.48 [0.20–0.58].

**Figure 2:**
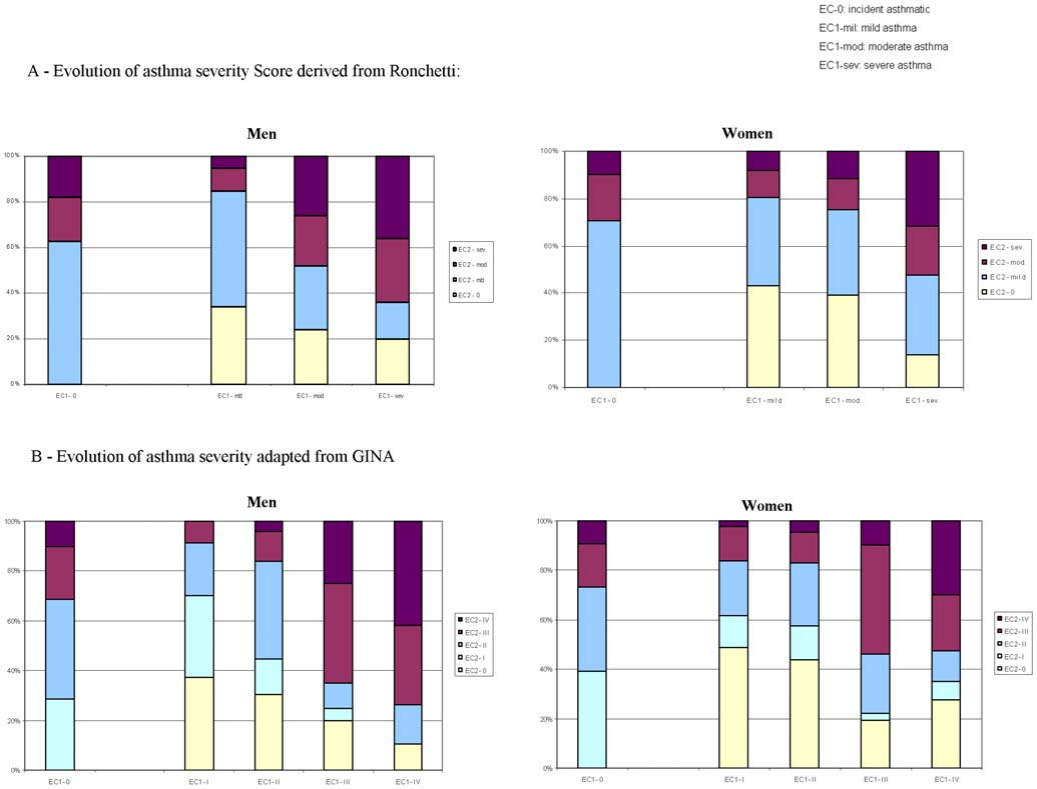
Distribution of asthma severity at EC2 in subjects without current asthma at EC1 (ECI-0) and in subjects with asthma, according to asthma severity at EC-1.

In contrast, gender differences were observed in the evolution of severity. Figure 2 shows the distribution of asthma severity between at follow-up, in incident asthmatics who had no asthma at the first survey, and in cases with asthma at baseline taking into account the severity of asthma at baseline.

There was no gender difference for severity in incident asthmatics. Using the Ronchetti score, the proportion of incident cases classified as having “mild” asthma was 62% in men and 69% in women (p = 0.7). There was also no gender difference in the distribution of asthma severity according to GINA for patients with incident asthma.

Using the Ronchetti classification (Fig. 2A), around 80% of subjects with mild asthma at baseline still had mild asthma, or had no “current asthma” at follow-up. According to both classifications, men with moderate-to-severe asthma at EC-1 were more likely than women to have moderate-to-severe asthma at EC-2: using Ronchetti-score, 53.3% of men vs. 36.2% of women with moderate-to-severe asthma at EC-1 were still moderate-to-severe at EC-2, (p = 0.03).

Using the GINA classification (Fig. 2B), 69.2% of men vs. 53.1% of women, with moderate-to-severe asthma at EC-1 were still moderate-to-severe at EC-2, (p = 0.09).

In contrast, in asthmatics with intermittent asthma at EC-1, the likelihood to develop moderate-to-severe asthma at EC-2 was higher in women (16.3% using GINA) than in men (8.8%), although the difference was not significant (p = 0.15). Using Ronchetti score, the likelihood to develop moderate-to-severe asthma at EC-2 was higher in women (20%) than in men (14.5%) (NS).

As regards hospitalization, respectively, 18%, and 22% of subjects with moderate persistent asthma, and severe persistent asthma were hospitalized for asthma during the previous year, without any significant difference between men and women.

As regards symptoms and lung function of subjects with severe asthma, 19.3% of women reported >12 asthma attacks in the preceding year or had FEV1<60% (“poor asthma control”) versus 30.2% of men. Conversely, 52.7% of men versus 37.1% of women with severe asthma were receiving a treatment that was “lower” than the one recommended for their level of severity.

Finally, in order to identify the factors related to change in asthma severity, we analyzed the subsample of subjects with moderate-to-severe asthma in the first survey (n = 127) and separated them into two groups: those who still had moderate-to-severe asthma in the second survey and those whose severity improved whatever the classification used, forty-seven subjects had an unchanged severity while 19 subjects were changed.

In women with moderate-to-severe asthma at baseline, sensitization to cat was associated with a higher risk of having no improvement in asthma severity at the second follow-up (59.3% vs 33%; p = 0.04). In contrast, in men three main factors were associated with no change in asthma severity: age at the second survey (more than 45 years) (52.8% vs 26%; OR: 0.76[0.59–0.98] p = 0.04), smoking history (66.6% vs 30.4%, OR: 0.85 [0.68–0.90] p = 0.006), and sensitization to molds (41.6% vs 9%; OR: 0.6[0.55–0.85] p = 0.008).

## Discussion

This investigation using two approaches is the first to study the evolution of asthma severity in a population-based longitudinal cohort of men and women. Whatever the classification, there was no difference in the overall distribution of asthma severity between men and women. However, investigating change in severity over time suggested that asthma severity might be more stable in men than in women. In particular, 69.2% of men with moderate-to-severe asthma at baseline still had moderate-to-severe asthma after a median follow-up of 8.7 years, whereas only 53.1% of women with moderate-to-severe asthma remained moderate-to-severe. In men with moderate-to severe asthma, factors significantly associated with the risk of remaining at the same level of severity were age, smoking status, and sensitization to mold. In women with moderate-to-severe asthma, only sensitization to cat was associated with no improvement in asthma severity at follow-up.

Various factors have been suggested to explain gender differences in asthma prevalence and incidence such as hormonal modulation[5], gender difference in hyper reactivity [18], [19], [20], preferential exposure of women to environmental triggers such as aeroallergens, and ventilatory response to PcO2[4].

However, it is unclear whether men or women generally suffer worse symptoms and greater deficits in lung function. Prevalence of asthma and allergies had been mapped by several international standardized studies[21], but little is known about the distribution of asthma severity in the general population from an epidemiologic point of view.

Assessment of severity is one of several keys to asthma management. Guidelines and consensus statements have been formulated by international panels of experts with regard to degree of severity and treatment[13], [15]. Asthma is characterized by various clinical symptoms such as episodic breathlessness, wheezing and chest tightness, with seasonal variability. According to the guidelines, severity may be judged by measurements of symptoms, lung function, and medication requirements. However, medication use might not be fully adequate for accurate severity classification, because it depends both on the practitioner and adherence to treatment by the patient, with the concomitant risk of under- or over-treatment. In addition, doses of inhaled corticosteroids may be subject to recall bias. Other factors, including comorbidity medical conditions that mimic asthma such as vocal cord dysfunction, may also lead to potential misclassification of patients with severe asthma [22].

Assessment of asthma severity in cohort studies has been mainly based on respiratory function [9]. Several studies have attempted to define severity scores using different approaches in children[16] and in adults[14]. Liard et al. proposed a classification using GINA guidelines [14] in a population of asthmatics recruited by chest physicians. By contrast, other authors included emergency visits or hospitalizations in severity assessment [16], [17]. Emergency visits and hospitalizations are usually used to describe the impact of the disease, which is related to asthma morbidity. It has been shown that acute asthma exacerbation is often life-threatening in patients who attend accident and emergency departments because of inadequate treatment, mainly due to under use of corticosteroids and inappropriate admission rates according to exacerbation severity[23]. However, including hospitalizations in the assessment of asthma severity is still a subject of debate. Recently, in a selected population of patients with difficult-to-treat asthma (TENOR), Miller et al. compared asthma severity assessment according to three methodologies[24]. They showed that classification of asthma severity on the basis of current asthma symptoms and lung function may be useful but not completely reflective of a patient's true asthma condition. Clinical assessment of asthma severity should consider a patient's medication use and consumption of health care resources for asthma exacerbation. They suggested that many adults with a history of moderate-to-severe allergic asthma in childhood had irreversible lung function deficits[25]. In addition, urgent care visits for asthma per year is now included in the criteria for defining severe/refractory asthma (20).

In our study, we found no gender difference in the overall distribution of asthma severity in the cross-sectional analyses, whatever the classification used. “In many studies investigating severe asthma, women are found to dominate the severe asthma group, with 59% to 82%(*) of severe asthmatics being women[22]. However, women also generally dominate the non-severe asthma group, and there are reports from other population-based studies showing no difference in the overall distribution of asthma severity between asthmatic men and asthmatic women [7]. In the ENFUMOSA study, however, the male to female ratio was still higher in the severe asthma group than in the group of asthmatics whose asthma was controlled by low doses of inhaled corticosteroids (male to female ration: 4.4∶1 vs. 1.6∶1, respectively; p<0.001)[3].

Differences in the procedure to identify and select asthmatic cases, and differences in severity definitions may explain the differences observed. In a recent study published by the TENOR group, females reported significantly greater healthcare utilization than males, significantly more asthma control problems and lower asthma-related quality of life. Despite their overall worse health outcomes, female subjects demonstrated better lung function, had similar treatment patterns, and showed no differences in physician-assessed asthma severity when compared with males [26]. In our study, moderate-to-severe asthma was more particularly characterized by a decrease in FEV1 (<60%pred) in men whereas in women it was associated with more intensive treatment. There was no significant difference between men and women with regard to hospitalization increases with asthma severity at the two surveys. These two results suggest that a significant gender difference exists for severity of the disease and for management (i.e. treatment). In our subjects, prevalence of remission was 23%. Remission diminished whereas asthma severity increased both in men and women. In the study by Sears et al.[9], remission was defined as the absence of wheezing after wheezing had been reported at two or more successive prior assessments. As in our study, similar remission rates (around 15%) were observed in men and in women. To our knowledge, little is known about gender difference in remission according to level of asthma severity.

Although no gender differences were observed in the overall distribution of asthma severity, differences were observed in the evolution of severity between the two surveys. In a cross-sectional analysis of the subjects with asthma from the ECRHS dataset at baseline, Zureik et al. showed that asthma severity was associated with sensitization to airborne moulds[17]. An other cross-sectional analysis showed that subjects with severe asthma at follow-up had poorer FEV1% predicted at baseline, poorer symptom control, higher IgE levels, and higher prevalence of chronic cough/mucus hypersecretion than patients with intermittent asthma. This study is the first to report on gender differences in changes in asthma severity, taking into account severity at baseline. Women were not more exposed to risk factors of change than men, suggesting that hormonal factors could explain changes in asthma severity during adulthood. However, another explanation could be that subjects whose severity changed could have had poorly controlled asthma at the first survey. Evaluation of asthma control is still difficult in clinical practice and is difficult to evaluate in epidemiological studies. Asthma is a complex, chronic disease varying from minutes to years. Our findings suggest that evolution of asthma severity is different in men and in women, and this difference is to be taken into account when investigating the variability of asthma severity in epidemiological studies.
